# Olfactory Dysfunction and Glaucoma

**DOI:** 10.3390/biomedicines12051002

**Published:** 2024-05-02

**Authors:** Valeria Iannucci, Alice Bruscolini, Giannicola Iannella, Giacomo Visioli, Ludovico Alisi, Mauro Salducci, Antonio Greco, Alessandro Lambiase

**Affiliations:** Department of Sense Organs, Sapienza University of Rome, Viale del Policlinico 155, 00161 Rome, Italy; valeria.iannucci@uniroma1.it (V.I.); alice.bruscolini@uniroma1.it (A.B.); giannicola.iannella@uniroma1.it (G.I.); giacomo.visioli@uniroma1.it (G.V.); ludovico.alisi@uniroma1.it (L.A.); mauro.salducci@uniroma1.it (M.S.); antonio.greco@uniroma1.it (A.G.)

**Keywords:** smell, olfaction, olfactory test, hyposmia, neurodegeneration, glaucoma, retinal ganglion cells, pseudoexfoliation syndrome, olfactory dysfunction, olfactory disorders

## Abstract

Background: Olfactory dysfunction is a well-known phenomenon in neurological diseases with anosmia and hyposmia serving as clinical or preclinical indicators of Alzheimer’s disease, Parkinson’s disease, and other neurodegenerative disorders. Since glaucoma is a neurodegenerative disease of the visual system, it may also entail alterations in olfactory function, warranting investigation into potential sensory interconnections. Methods: A review of the current literature of the last 15 years (from 1 April 2008 to 1 April 2023) was conducted by two different authors searching for topics related to olfaction and glaucoma. Results: three papers met the selection criteria. According to these findings, patients with POAG appear to have worse olfaction than healthy subjects. Furthermore, certain predisposing conditions to glaucoma, such as pseudoexfoliation syndrome and primary vascular dysregulation, could possibly induce olfactory changes that can be measured with the Sniffin Stick test. Conclusions: the scientific literature on this topic is very limited, and the pathogenesis of olfactory changes in glaucoma is not clear. However, if the results of these studies are confirmed by further research, olfactory testing may be a non-invasive tool to assist clinicians in the early diagnosis of glaucoma.

## 1. Introduction

The sense of smell is one of the human senses that enables the perception of odors or scents in the environment. Although often neglected in medical practice, an olfactory dysfunction significantly impacts the quality of life [1]. Hyposmia or anosmia reduces the enjoyment of food, social relationships, and work skills. In addition, alterations in the sense of smell can expose people to the risk of damage from fumes, toxic substances, and spoiled food. 

In recent years it has been shown that olfactory dysfunction may be an early sign of major neurodegenerative diseases such as Parkinson’s and Alzheimer’s [2,3,4]. In addition, it seems that the deterioration of olfactory function could be associated with the progression of these neurodegenerative disease and early cognitive impairment [1,5]. According to this evidence, Knight and colleagues reported that better olfactory performance is associated with a lower risk of progression of cognitive impairment and is a predictor of longevity [6].

The retina and optic nerve are highly specialized neural structures and direct extensions of the central nervous system. The interdependence of the brain, optic nerve, and retinal tropism has been well documented [7], and thinning of the retinal nerve fiber layer (RNFL), assessed with optical coherence tomography (OCT), has been confirmed in many neurodegenerative diseases, such as Alzheimer’s [8,9], Parkinson’s [10], and multiple sclerosis [11]. 

Glaucoma is an optic neuropathy characterized by visual field defects and changes in the optic nerve head and nerve fiber layer; it is the leading cause of irreversible bilateral blindness, affecting approximately 80 million people worldwide [12].

Glaucoma shares some common pathogenetic mechanisms, particularly with Alzheimer’s disease, such as mitochondrial dysfunction, neuroinflammation, and oxidative stress [13,14,15,16]. Over the years, evidence has shown that glaucoma should not be considered a purely ophthalmologic disease because it damages not only the optic nerve but also other brain structures involved in vision [17,18].

Could glaucoma, as a neurodegenerative disease of the visual system, be associated with early neurological olfactory damage, as well as Alzheimer’s and Parkinson’s diseases?

This review analyzed this medical hypothesis and reported the current scientific evidence on these two potentially related topics.

## 2. Smell Dysfunction Causes

Several clinical conditions can cause olfactory dysfunction [1].

- Upper respiratory infections;
- Nasal and sinus conditions such as sinusitis, nasal polyps, or inflammation of the nasal passages can interfere with the ability to smell by blocking airflow or affecting olfactory receptors;
- Head trauma, including injuries to the head, especially to the frontal lobes or the area around the nose, can damage the olfactory nerves and cause loss of smell;
- Aging: as people age, there may be a natural decline in the sense of smell due to changes in the olfactory system;
- Medications: some medications, including certain antibiotics, antidepressants, and antihypertensives, can cause olfactory dysfunction as a side effect;
- Toxic chemical exposure: exposure to certain chemicals or toxins, such as solvents, pollutants, or heavy metals, can damage the olfactory system and cause loss of smell;
- Genetic factors: in rare cases, genetic conditions can affect a person’s ability to smell.
- Neurological conditions: neurological diseases, such as Alzheimer’s, Parkinson’s, multiple sclerosis, or epilepsy, can affect the sense of smell.

The association between neurodegenerative diseases and smell issues is well established in the literature [2,3,4,5,11,19,20,21,22,23,24,25,26,27]. It is estimated to be present in 90% of patients with early Parkinson’s disease (PD) and 85% of patients with early Alzheimer’s disease (AD) [28]. Olfactory dysfunction has been reported as a prodromal symptom, which is often given little clinical relevance. Hyposmia is already present in mild cognitive impairment and tends to worsen as the disease progresses; odor identification is the most impaired among the olfactory functions in these patients [3,5,19,29].

In PD, hyposmia is one of the first non-motor signs of the disease [28,30,31], and olfactory hallucinations (phantosmia) are reported in up to 21% of cases [32]. In addition, the olfactory threshold has been proposed as a test to differentiate Lewy bodies dementia (LBD) from Parkinson’s dementia, as patients with LBD have a much lower olfactory threshold [33].

Although olfaction is compromised in both AD and PD, Rahayel and colleagues note that olfactory identification and discrimination, which require higher cognitive functions, are more impaired in AD. In contrast, the olfactory threshold is lower in PD [27].

The pathophysiology of olfactory dysfunction in neurodegenerative diseases is unclear.

In an immunocytochemical study, Brozetti et al. found that molecules classically associated with neurodegeneration, such as α-synuclein, β-amyloid, and hyperphosphorylated tau, were present in the olfactory neuroepithelia of healthy subjects. The researchers hypothesize that these proteins are misfolded in response to an unknown stimulus and then transported to the olfactory bulb, gradually forming pathological aggregates. This process would cause the early olfactory impairment observed in diseases such as Alzheimer’s and Parkinson’s [34].

Furthermore, smell is not only impaired in AD and PD. In multiple sclerosis, olfactory dysfunction has a prevalence of 27.2% [11], and there appears to be a correlation between olfactory bulb volume reduction and hyposmia [35]. According to several meta-analyses, many other neuropsychiatric disorders affect the sense of smell (Table 1). Specifically, olfactory discrimination, impaired in schizophrenia but not in frontotemporal dementia, may contribute to the differential diagnosis between these two disorders [36].

### Olfactory Dysfunction Evaluation

Different tests have been developed to test olfactory function. Electrophysiological tests (olfactory evoked potentials, OEPs) are mainly used for research and legal purposes [37]. In clinical practice, the sense of smell can be assessed with relatively simple, non-invasive, and inexpensive tests. 

The most widely used and established tests in clinical practice are the Sniffing Stick Test (SST) and the University of Pennsylvania Smell Identification Test (UPSIT) [38].

The SST is a pen-like odor delivery device, designed and developed by Kobal and Hummel [39,40]. This test assesses all three components of olfactory function, expressed in a final score (threshold, discrimination, and identification Score, TDI score).

Odor identification tests ask the patient to identify a series of odors, one at a time, by answering a multiple-choice questionnaire. Odor discrimination tests the individual’s ability to identify which of the three odors presented is different from the other two. The odor threshold is a measure of the lowest concentration of an odorant substance that the subject can detect using serial dilutions [41,42]. 

The UPSIT (University of Pennsylvania Smell Identification Test) is a standardized test to evaluate the olfactory function. It is a scratch-and-sniff test that presents various odors in a multiple-choice format to identify the type of smell.

The UPSIT is widely used in clinical settings to assess the ability to identify different smells and to detect changes or deficiencies in the sense of smell. It helps in diagnosing various conditions related to olfactory dysfunction, such as anosmia (complete loss of smell), hyposmia (reduced sensitivity to smell), or specific smell disorders (Table 2).

This test provides a quantitative measure of olfactory function, aiding doctors in determining the degree of impairment and monitoring the olfactory dysfunction of smell over time.

## 3. Glaucoma and Neurodegeneration

Glaucoma is a heterogeneous group of diseases clinically characterized by specific patterns of visual field loss and optic nerve head damage. Diffuse damage to highly specialized neural structures and an insidious onset that causes noticeable symptoms only in the late stages of the disease, make glaucoma the leading cause of irreversible blindness worldwide [12]. Glaucoma is often associated with high intraocular pressure (IOP), which is one of the major risk factors for developing the disease and the only therapeutic target for medical and surgical treatment. For this reason, glaucoma has traditionally been considered a purely ophthalmologic disease. Today, there is increasing evidence [17,18,44,45,46] that glaucoma should be considered a multifaceted neurodegenerative disease in which IOP is only one piece in a complex mosaic. 

Specifically, apoptosis of the retinal ganglion cell (RGC) is the hallmark of glaucoma, but it has been widely shown that other brain structures associated with vision are also involved [17,47].

The RGC is a highly specialized neuron whose cell soma is located in the inner layers of the retina. The axons of these cells converge to form the optic nerve, the chiasm (where they partially cross over), and the optic tract and then form synapses in the lateral geniculate nucleus. The primary visual pathway then continues through optic radiation to the primary cerebral cortex, providing the conscious experience of vision.

Along the retino–geniculo–cortical pathways, visual stimuli are divided into two main systems: the magnocellular and parvocellular systems. The magnocellular system has a faster response time and seems to be specialized for visuospatial perception, motion, and stereopsis, allowing for rapid detection and localization of a moving object in the visual field. The parvocellular system, on the other hand, has a slower response time and provides discriminative cues such as the shape, color, and contrast of the observed object. This allows the object to be examined, recognized, and categorized [17,18]. This topographic map is present in the retina, is replicated in the lateral geniculate nucleus, and is maintained all the way to the visual cortex.

There is evidence in both animal models and humans that glaucoma damages both the parvocellular and magnocellular pathways, but there is no agreement on which one is more impaired [17,18].

Loss of RGCs and their axons causes atrophy of the optic nerve head and inner retinal layers, which can be detected by clinical examination and optical coherence tomography (OCT), and also appears to affect the posterior brain structures of the visual system.

Damage to the posterior visual pathways is suggested by increased latency and reduction of the electrical signal as measured by visual evoked potentials (VEPs) [47].

This widespread cerebral damage has been confirmed by nuclear magnetic resonance (MRI) imaging studies. MRI morphometric analyses have documented that neurodegeneration in glaucoma patients affects not only the optic nerve, chiasm, and optic tract, as might be expected, but also the lateral geniculate nucleus of the thalamus, the optic radiations, and the visual cortex [45,46] Specifically, in glaucoma, trophism and function of the primary visual cortex, as assessed by functional MRI, correlate with both RNFL thickness and perimetric defect [44].

This evidence suggests that the trophism of neurons involved in visual pathways is interdependent, as has been demonstrated in other neurodegenerative diseases, and that atrophy in glaucoma spreads to all the cerebral structures involved in vision. This would be true both from the retina to the brain (anterograde neurodegeneration) and vice versa, from the brain to the retina (retrograde neurodegeneration), via trans-synaptic degeneration [18].

The causes of RGCs loss are not fully understood. 

According to the mechanical theory, ocular hypertension is thought to disrupt axoplasmic transport by mechanically compressing the optic nerve bundle through the pores of the lamina cribrosa, [48] inducing apoptosis in RGCs. The lamina cribrosa is a diaphragm between the intraocular and subarachnoid compartments, and the delta between intraocular and intracranial pressure, called the translaminar cribrosa pressure (TLCP) gradient, appears to be involved in the pathogenesis of glaucoma. Thus, not only increased IOP but also decreased cerebrospinal fluid pressure can mechanically deform the lamina cribrosa [48] and damage the optic nerve head.

As highly specialized neurons, RGCs are particularly susceptible to hypoxic injury. Therefore, the ischemic theory suggests that impaired blood flow may promote neurodegeneration in glaucoma; accordingly, increased expression of hypoxia-induced factor 1alpha (HIF-1alpha) has been found in retinal areas corresponding to perimetric scotomas [49]. In addition to the mechanical and ischemic theories, excitotoxicity, oxidative stress, and neuroinflammation also play a role in neurodegeneration [48].

In excitotoxicity, there is an alteration in synaptic homeostasis with excessive glutamate release from the presynaptic neuron and calcium ion accumulation at the postsynaptic neuron. Excess calcium would activate lytic enzymes (lipase, endonuclease, and protease) and nitric oxide synthetase, which would induce cell apoptosis.

Mitochondrial dysfunction and the reduction of antioxidant enzymes also appear to play a role in glaucoma, as does neuroinflammation mediated by astrocytes, microglia, and Müller cells [48].

Glaucoma has a multifactorial complex etiology, and the above pathogenetic mechanisms have been described in other neurodegenerative diseases, particularly Alzheimer’s disease [13,14,15]. In fact, cohort studies have shown an overlap between the risk of developing glaucoma and Alzheimer’s disease (Bayer et al. showed a 25.9% prevalence of glaucoma in AD) [50].

Apoptosis is the common step that leads to loss of function, mainly affecting the limbic system in AD and the visual pathways in glaucoma [13]. 

In line with these data, glaucoma therapy has now fully embraced neuroprotective molecules, such as citicoline [7,51,52], that have been used in Alzheimer’s disease therapy [53,54], and other neurotrophins have been studied in glaucoma research [55,56,57]. 

As previously mentioned, glaucoma is classically associated with ocular hypertension, one of the main risk factors for developing the disease. 

However, the relevance of primary neurodegeneration is particularly evident in cases of normal tension glaucoma (NTG), where the intraocular pressure (IOP) lies within the normal range, but the visual field loss often progresses independently, suggesting a pressure-independent mechanism of neurodegeneration.

The pathogenesis of NTG remains unclear, but several mechanisms have been proposed, including ischemic theory and increased TLCP gradient, which may contribute significantly to neurodegeneration in these patients [58,59].

Specifically, microvascular endothelial dysfunction and abnormal vasoreactivity in response to the sympathetic autonomic nervous system (PVD, primary vascular dysregulation, according to Flammer and colleagues) have been observed, supported by an imbalance between nitric oxide (NO, vasodilator) and endothelin-1 (ET-1, vasoconstrictor) [60]. Reynaud’s phenomenon, migraine, and silent cardiovascular and cerebrovascular diseases are often associated with normal tension glaucoma [58,59]. Accordingly, Takahashi et al. found a correlation between abnormal nailfold and optic disc vasoconstriction in response to the cold-water provocation test in NTG patients [61].

NTG appears to be even more related to Alzheimer’s disease than glaucoma associated with ocular hypertension. In fact, Tamura et al. found a high prevalence of glaucoma (23.8%) in AD compared to healthy subjects (*p* = 0.0002), with no differences in intraocular pressure [62]. In a 13-year retrospective cohort study, Chen et al. found an increased risk of Alzheimer’s disease in normal tension glaucoma (Hazard Ratio 1.52) in the Taiwanese population, supporting the link between these diseases [63].

Another shared feature between glaucoma and AD is the abnormal deposition of proteinaceous aggregates [13]. Plaques of beta-amyloid and tangles of hyperphosphorylated tau protein are the hallmarks of Alzheimer’s disease, and pseudoexfoliative glaucoma (PXG) is associated with diffuse deposition of proteinaceous material (pseudoexfoliation syndrome, PXS).

In PXS, these fibrillar deposits can be found in ocular and extraocular tissues, such as the vascular endothelium, skin, heart, liver, lungs, kidneys, meninges, and gallbladder. The ocular complications of PXS include glaucoma and zonular weakness with possible lens dislocation following trauma or surgery [64].

According to the literature, pseudoexfoliation syndrome may be a marker of neurodegeneration and share features with Alzheimer’s disease [65]. In fact, both PXS and AD are age-related disorders and involve abnormal protein misfolding and aggregation [13,64]. Furthermore, apolipoprotein E (APOE) and amyloid, which play a key role in the pathogenesis of Alzheimer’s disease [13,15,63], were also found in the fibrillar material of PXS [64].

Glaucoma and Alzheimer’s are very different diseases, and their pathogeneses are still unclear, but their common features have attracted the attention of researchers.

Could there be a common pathogenetic mechanism that affects both the olfactory and visual systems at the same time, causing glaucoma patients to experience early loss of smell as described in other neurodegenerative diseases?

## 4. Smell Function and Glaucoma

Unfortunately, this topic has not been widely studied and reported in the literature.

After a literature search, just three articles analyzing glaucoma and olfactory dysfunction were identified and considered (Table 3).

Mozaffarieh and colleagues investigated olfaction in normal tension glaucoma (NTG) [66]. Thirty-six patients with NTG and 36 healthy controls matched for age and sex were enrolled; in both groups, half of the subjects had signs compatible with vasospasm, such as migraine, tinnitus, hypotension, and cold extremities. Olfaction was assessed by self-report and SST. Subjects with vasospasm were significantly better at identifying odors than the others, either by self-report or SST (*p* < 0.001), whether they were glaucomatous or healthy subjects. The difference remained statistically significant even after the results were adjusted for age. This study only uses the 12-item identification test in the olfactory dysfunction evaluation. This is a substantial limitation. The authors speculate that subjects with PVD, who would already have differential gene expression of ATP-binding cassette (ABC) proteins [67], may also have a different gene expression of odorant-binding proteins, which would result in increased odor perception. 

**Table 3:** Smell function and glaucoma.

Authors	*n*	Smell Test	Outcomes
Mozaffarieh, M. et al. (2010) [66]	36 NTG patients and 36 healthy controls; half participants in both groups had PVD.	SST	In both groups, subjects with PVD had better smell outcomes (*p* < 0.001)
Gugleta, K. et al. (2010) [68]	30 POAG patients	SST	Olfactory threshold was significantly lower in POAG than healthy controls (*p* = 0.01)
Dikmetas, O. et al. (2022) [69]	20 POAG patients		
	20 PXG patients	SST	Complex olfactory abnormalities that affected threshold, discrimination, and identification differently in each group. The PXG group showed the worst TDI score.
	20 PXS patients		

NTG = normal tension glaucoma; POAG = primary open-angle glaucoma; PXG = pseudoexfoliative glaucoma; PXS = pseudoexfoliative syndrome; PVD = primary vascular dysregulation; SST = Sniffin Stick test; TDI score = threshold discrimination identification score.

Subsequently, Gugleta et al. enrolled 30 patients with primary open-angle glaucoma (POAG) and compared olfactory performance with a healthy control group [68]. While the TDI score did not differ significantly between the two groups, the olfactory threshold was significantly lower in POAG than in healthy controls (*p* = 0.01). Again, patients with evidence of vasospasm, as assessed by a cold extremity and nail capillaroscopy, had a better olfactory threshold compared to POAG patients without vasospasm (*p* = 0.036).

In a cross-sectional study published in 2022, Dikmetas and colleagues investigated olfactory function in POAG and PXG [69]. The researchers included 20 patients with POAG, 20 with PXG, and 20 subjects with PXS without glaucoma. Olfactory performance was assessed using the SST, and significant differences were found between the groups. 

Specifically, odor identification was significantly lower in POAG compared to the control group (*p* = 0.01) and in PXG compared to all others (*p* < 0.000 for POAG and PXS *p* < 0.02 for healthy subjects).

Odor discrimination was significantly impaired in subjects with PXG compared to the others (*p* < 0.000 for each group).

Finally, patients with POAG and PXG had a statistically significant impairment in olfactory threshold compared to healthy controls (*p* = 0.033 and *p* = 0.001, respectively). 

According to the TDI score, smell function was more compromised in PXG. Olfactory sensitivity was also impaired in POAG compared to healthy subjects and in PXS compared to POAG and healthy subjects.

These data may support that, in glaucoma, as in other neurodegenerative diseases, complex olfactory abnormalities mainly affect odor sensitivity. Furthermore, different types of glaucoma appear to be associated with different olfactory dysfunctions. The authors hypothesize that a possible link between olfactory dysfunction and glaucoma may be related to abnormal accumulation of tau protein, which has been demonstrated in both murine models of Parkinson’s disease with olfactory dysfunction [70] and murine models of glaucoma [71].

## 5. Discussion 

The olfactory function has been studied in many neurodegenerative diseases, mainly Alzheimer’s and Parkinson’s. However, there is little data on olfaction in glaucoma, a neurodegenerative disease of the visual system. Nevertheless, the available data are interesting.

First, not all types of glaucoma affect olfactory function similarly. Mozaffarieh and Gugleta found hyperosmia in their sample, while Dikmetas found reduced olfactory function in patients with PXG compared with PAOG and in POAG compared with the healthy control group [66,68,69].

PXG develops in the context of a PXS, and NTG is often associated with blood-flow abnormalities in response to stimulation of the sympathetic nervous system (PVD, primary vascular dysregulation, according to Flammer and colleagues) [60]. 

The significance of hyperosmia in patients with primary vascular dysregulation and its possible association with NTG can only be cautiously speculated. The available data suggest that patients with PVD are more likely to experience hyperosmia and may be more prone to develop NTG, and a possible link between hyperosmia, vasospasm, and neurodegeneration should be further investigated.

To summarize the results of these studies, certain predisposing conditions to glaucoma, such as pseudoexfoliation syndrome and primary vascular dysregulation, may possibly induce olfactory changes that can be measured with the Sniffin Stick test. 

However, when the olfactory performance of PXS patients is compared with those who developed glaucoma, the latter have worse olfactory function.

In addition, patients with POAG appear to have worse olfaction than healthy subjects, although this is less pronounced than in people with PXG.

More evidence is needed to update clinical practice and incorporate olfactory testing into glaucoma management, as these studies have some limitations. All three studies have small sample sizes. Mozaffarieh and Gugleta focused on PVD, while Dikmetas studied PXG, so the results of the three studies are not fully comparable. None of the studies correlated olfactory function with the visual field, and the possible effects of glaucoma medical therapy on olfaction are not explained. Finally, although the results of these studies are statistically significant, there are no clear hypotheses to justify them. It could be speculated that olfactory neural pathways may be particularly susceptible to non-specific neurodegeneration. This may explain the evidence of olfactory dysfunction in a wide range of neuropsychiatric disorders and possibly in glaucoma.

Although there are few studies on this topic, the possibility that olfaction may be impaired in glaucoma remains attractive and is based on two well-established data in the literature, namely olfactory dysfunction is an early marker in a variety of neurodegenerative diseases, and glaucoma is not limited to the eye but is a complex neurodegenerative disease of the visual system. 

Expansion of the sample size and refinement of the protocol may overcome this lack of evidence. If these speculations are confirmed in further research, olfactory testing may be useful in the future to facilitate early diagnosis of glaucoma, e.g., by screening patients with PXS and patients with Raynaud’s phenomenon or nail capillaroscopy alterations. 

The SST can quantitatively measure olfactory dysfunction. In glaucoma, there appear to be complex olfactory modifications that may alter the olfactory threshold, discrimination, and identification to variable degrees. The TDI score gives an idea of the overall olfactory dysfunction but also provides a precise characterization of each of the olfactory functions. This, combined with the quick administration (15–20 min), makes the SST the most interesting test to assess olfactory function in glaucoma.

As olfactory testing is inexpensive and non-invasive, and glaucoma has a high social burden, it may be worthwhile to investigate further the possible links between the visual and olfactory systems. It may be interesting to assess whether there is a correlation between visual, cognitive, and olfactory function. Furthermore, recent works have correlated pseudoexfoliation syndrome with neurosensory hearing loss [72] and possibly glaucoma [73]. These data suggest that PXS may correlate with multisensory impairment (hyposmia, glaucoma, and hearing loss), opening the possibility of a multidisciplinary approach to screening for sense-organ disorders, which would likely have a positive impact on the quality of life of these patients.

## Figures and Tables

**Table 1:** Smell function involvement in neuropsychiatric diseases.

Alzheimer’s disease and Mild cognitive impairment	Jung et al. 2019 [5]. Roalf et al. 2017 [19]. Rahayel et al. 2012 [27]. Kotecha et al. 2018 [3]. Mesholam et al. 1998 [2].
Parkinson’s disease	Mesholam et al. 1998 [2]. Rahayel et al. 2012 [27] Sui et al. 2019 [4]. Lyu et al. 2021 [23]. Alonso et al. 2021 [20] Trentin et al. 2022 [21]
Multiple sclerosis	Mirmosayyeb et al. 2022 [11]
Epilepsy	Khurshid et al. 2019 [22]
Rapid Eye Movement Sleep Behavior Disorder	Lyu et al. 2021 [23].
Autism-spectrum disorders	Crow et al. 2020 [24]
Obsessive–Compulsive Disorder	Crow et al. 2020 [24]
Severe anorexia nervosa	Mai et al. 2020 [25]
Schizophrenia	Carnemolla et al. 2020 [36]
Frontotemporal dementia	Carnemolla et al. 2020 [36] Kamath et al. 2019 [26]

**Table 2:** Smell dysfunctions [43].

Term	Definition
Dysosmia	General term for smell dysfunction
Quantitative smell dysfunction	
Anosmia	Sense of smell is absent or almost absent, with an impact on daily life activity
Hyposmia	Composite TDI score* < 10th percentile
Normosmia	Composite TDI score* > 10th percentile
Hyperosmia	Composite TDI score* > 90th percentile
Qualitative smell dysfunction	
Olfactory intolerance	Intolerance to common odors, unconfirmed with smell tests.
Parosmia	Olfactory dysperception in the presence of a real odor stimulus
Phantosmia	Olfactory dysperception in the absence of a real odor stimulus

* TDI-score: Threshold Discrimination Identification score.

## Data Availability

No new data were created or analyzed in this study. Data sharing is not applicable to this article.
